# Whole-Colony Dynamic Energy Budget Model for Bumble Bees: Assessing the Impact of Wildflower Patches on Crop Pollination

**DOI:** 10.1007/s11538-025-01448-8

**Published:** 2025-05-21

**Authors:** Pau Capera-Aragones, Joany Mariño, Amy Hurford, Rebecca C. Tyson, Eric Foxall

**Affiliations:** 1 Institute of Chemistry, The Hebrew University of Jerusalem, Jerusalem, Israel; 2 CMPS Department (Mathematics), University of British Columbia Okanagan, Kelowna, Canada; 3 Department of Biology, Memorial University of Newfoundland, St. John’s, Canada; 4 Department of Mathematics and Statistics, Memorial University of Newfoundland, St. John’s, Canada; 5 Department of Internal Medicine B, University Medicine Greifswald, Greifswald, Germany; 6 Department of Chemistry, Georgia Institute of Technology, Atlanta, USA

**Keywords:** Dynamic energy budget, maximum entropy principle, bumble bee, pollination services, sustainable agriculture.

## Abstract

Bumble bees are important pollinators of many crops around the world. In recent decades, agricultural intensification has resulted in significant declines in bumble bee populations and the pollination services they provide. Empirical studies have shown that this trend can be reversed by enhancing the agricultural landscape, for example, by placing wildflower patches adjacent to crops. Despite the empirical evidence, the mechanisms behind these positive effects are not fully understood. Theoretical studies, in the form of mathematical or computational models, have proven useful in providing insights, but the complexity of the underlying system means that certain factors remain unexplored. In this work, we build a unique model coupling a whole-colony Dynamic Energy Budget (DEB) approach for population dynamics to a Maximum Entropy (MaxEnt) principle formulation for the spatial distribution of foraging bees. The use of a DEB to asses whole-colony energy budgets, and its coupling to a spacial model is novel. The use of MaxEnt to predict foraging spatial distributions is still in its early stages, and our work highlights its potential to advance and expand upon the traditional assumptions of the Ideal Free Distribution. We use the developed model to asses the possible benefits and drawbacks of planting wildflower nearby crops for crop pollination services. We answer questions of when should wildflowers bloom, how many should we plant, which type of wildflowers, and where should we place them.

## Introduction

Wild pollinators, such as bumble bees, provide important pollination services to crops around the world (Winfree et al. 2009; Porto et al. 2020). It is therefore concerning that wild bee populations are in decline (Biesmeijer et al. 2006; Potts et al. 2005; Vanbergen et al. 2013), along with the pollination services they provide (Turo et al. 2024). Agricultural intensification, characterized by the high use of pesticides and fertilizers, increases in farm size, low proportion of natural habitat in the landscape, and simplified crop rotation (Suhardiman 2016; Xie et al. 2019), is considered to be the major reason for pollinator habitat loss and the widespread decline of farmland biodiversity (Robinson and Sutherland 2002).

Several empirical studies have shown that the addition of natural habitat adjacent to pollinator-dependent crops is an effective strategy to prevent population and diversity declines in wild pollinators (Blaauw Brett and Isaacs 2014; Buhk 2018; Carvalheiro et al. 2011). A larger and more diverse pollinator community can also lead to increased pollination services to crops (Centeno-Alvarado et al. 2024; Blaauw Brett and Isaacs 2014). In Blaauw Brett and Isaacs (2014), the benefits of natural habitat enhancement were observed in the first year following plantation, and increased significantly over the course of the 4-year study.

Despite the empirical evidence in favour of using wildflower enhancements to increase crop pollination, the costs of these management strategies together with the lack of clarity on the optimal location, quantity, flower type, or flowering time of the wildflower enhancements diminishes the practical effectiveness of these strategies (Albrecht et al. 2020; Drummond and Hoshide 2024). Models studies can be very useful tools for exploring this complex system (Rouabah et al. 2024).

Previous modelling studies focused largely on the bumble bee population response to wildflower enhancements. Most show that wildflower patches can increase local bumble bee populations (Twiston-Davies et al. 2021; Haussler et al. 2017), generally resulting in increased wild bee pollination services (Haussler et al. 2017). However, some empirical (Delphia et al. 2022) and modelling (Nicholson et al. 2019) work indicate that this pattern may not always hold. Recent modelling studies have provided some useful insights into this conundrum, pointing to several factors that affect the effectiveness of wildflower enhancements. These include timing of the crop and wildflower bloom (Carturan Bruno et al. 2023), several properties of wildflower patches including size, density, and nutritional quality, all relative to the crop (Capera-Aragones et al. 2021, 2022), the relative positions of nest, crop, and wildflower sites (MacQueen et al. 2022; Capera-Aragones et al. 2021), and the intensity with which the landscape is farmed (Carturan Bruno et al. 2023; Nicholson et al. 2019; Capera-Aragones et al. 2021). Any one of these factors, if not satisfied, can reduce the positive effects of wildflower enhancements.

Given the large number of factors influencing wildflower patch effectiveness, modelling approaches are crucial. Existing models each address several components of the system, but assumptions are necessarily made to simplify each model and keep its study tractable (Rouabah et al. 2024). It is clear, however, that all of the included and omitted factors can be important, depending on the context, and may interact in unexpected ways (Sterman 2002; Willcox et al. 2024). Furthermore, the tools and assumptions used to build each model mean that simulations can answer certain questions, but not others (Rouabah et al. 2024), and so diversity in the modelling effort is critical (Odenbaugh 2005).

Here, we present a novel modelling approach where population growth depends explicitly on the energy needs of the colony, and functions within a spatially-explicit differential equation framework. That is, we include multi-year population dynamics, via an accounting of the energy needs of the colony, with the bees foraging in spatially explicit two-dimensional landscapes that are heterogeneous in time and space. Furthermore, the model is formulated using differential equations, rather than agent-based computation, making it less computationally expensive and more explainable.

The model developed here combines Dynamic Energy Budget (DEB) theory with the Maximum Entropy Principle (MaxEnt) (Capera-Aragones et al. 2023). It is the first to (1) apply DEB at a colony level and (2) to couple it to a spatial model. The DEB component predicts the population dynamics of the colony, and the MaxEnt component predicts the spatial distributions of foragers. Below, we briefly describe each of these approaches.

DEB modelling is a mechanistic approach for predicting behaviour through an accounting of the main energy fluxes in the system (Nisbet et al. 2012), and has been used to study a wide range of organisms (Mariño 2019; Pouvreau et al. 2006; Van Haren and Kooijman 1993; Humphries et al. 2004; Lavaud et al. 2021), including bumble bees (Kenna et al. 2019). Existing models are overwhelmingly based on the energy budgets of *individuals* (Nisbet et al. 2012). We modify this standard by applying the DEB approach to the entire *colony*, which allows us to obtain colony-level insights into population behaviour.

The MaxEnt approach uses the concepts of entropy and energy to predict forager distributions. The approach is computationally less intensive than most current methods used to predict spatial distributions in that (1) the number of individuals considered does not affect simulation performance as in individual-based models, and (2) the spatial scale considered does not affect simulation performance as in partial-differential equation models. It is thus a highly efficient and flexible way to predict forager distributions in a spatially inhomogeneous landscape (Capera-Aragones et al. 2023; Phillips et al. 2006). To date, MaxEnt has chiefly been used to understand species presence data at the landscape scale (O’Dwyer et al. 2017; Phillips et al. 2006), and is not commonly used as a component of dynamical models in Ecology.

Our DEB-MaxEnt model both accounts for important ecological properties of the flower-pollinator system and is mathematically simpler than other models (Becher et al. 2018; Becher and Grimm 2016; Carturan Bruno et al. 2023). It allows us to link the spatial arrangement of the landscape, in terms of agricultural intensity and the location and abundance of wildflower patches, to the flow of energy in and out of the colony via bee foraging. Finally, the computational simplicity of the model makes it easy to explore, at a fine resolution, the simultaneous effects of variation in the many environmental parameters relevant to the system. With this new perspective, we explore in detail how population growth and decline occur in response to landscape-induced changes in the energy gains and deficits of the colony, and the consequent effects on crop pollination services.

## Model and Methods

Dynamic Energy Budget (DEB) models provide a quantitative framework to dynamically describe the energy and mass budgets of living organisms at the individual level, based on assumptions about energy uptake, storage, and utilization of various substances (Sousa et al. 2008; Jusup 2017; van der Meer 2006). DEB models use thermodynamic principles such as conservation of mass, energy and time, and relationships between the surface and the volume of the individuals to link different levels of biological organization, i.e. from cells to individuals to populations (Kooijman 2010; Sousa et al. 2010).

The theory has been successfully applied to over 1000 different species (Marques et al. 2018) with applications in general ecology, conservation, aquaculture, and ecotoxicology (Lavaud et al. 2021). In our work, we develop a DEB-based model for wild bees pollinating a crop. In contrast to traditional DEB models, we consider the individual to be the whole colony, and apply the DEB approach to that “super-organism”. This framing means that the energy fluxes in our model are tracked at the level of the colony and not the individual bees, although the two are of course connected. The energy uptake, for example, is proportional not to the nectar collected by an individual bee, but to the total nectar collected by all bees, which is related to their foraging spatial distribution on the landscape. Applying the DEB framework to the whole colony increases the predictive capabilities of the model at the colony level without making the model significantly more complicated, and allows us to obtain insights into the energy budget and management of the whole colony. Individual bees forage chiefly according to the needs of the colony, and not their individual needs (Hagberry 2011; Hendriksma et al. 2019). A DEB that describe the energy budget of individual bees and ignores colony needs can not predict the foraging decisions of individuals, but a DEB that describe the energy budget of the whole colony can.

The spatiotemporal distribution of foragers, i.e., where and when individual bees are able to find resources, has a strong effect on the energy fluxes of the colony. We therefore couple our DEB model to a novel spatially implicit model based on the application of the Maximum Entropy Principle recently developed by Capera-Aragones et al. (2023) and summarized in Sect. 2.4. The spatial model can be understood as an extension of the Ideal Free Distribution (Fretwell 1969) that mechanistically accounts for travelling costs, foraging efficiency, and resource depletion. Using this approach to compute the spatial distribution of foragers keeps our model simple, while at the same time allows us to gain qualitative insights into the interaction between foragers and the landscape.

### Model Overview

We begin our presentation of the model with a brief overview of the model’s structure. The full model has two components, the dynamic energy budget and the spatiotemporal distribution of foragers and resources. Each is characterized by several variables:

1. The dynamic energy budget component has four state variables: Nectar reserves (N), Pollen reserves (P), Structural energy (V), and Number of colonies (R).
2. The spatiotemporal distribution of foragers and resources is described by two sets of vectors, indexed by the patch number *i*:
  - the nectar and pollen forager distributions $(\rho _i^N)$ and $(\rho _i^P)$, and
  - the environmental variables that give the amount of pollen and nectar resources in patch *i* of the landscape, $(F_i^N)$ and $(F_i^P)$.

The model variables and parameters (the latter are all constant over time) are summarized in Tables 1 and 2.

**Fig. 1:**
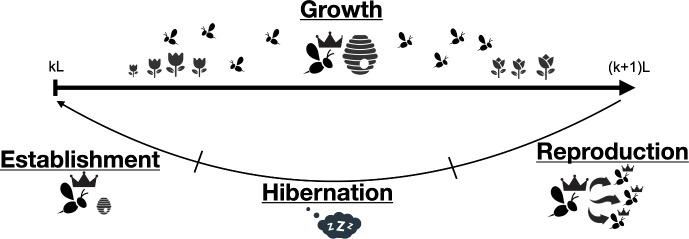
Schematic representation of the life cycle of the bumble bee colony (time periods not to scale). Four phases are represented; colony growth, reproduction, hibernation, and colony establishment. In our model, the first phase is described using a continuous-time model, the next three phases are simplified into one discrete-time jump. The year number is given by *k*, and *L* is the length of the growth phase

Bumble bee colonies exhibit several distinct phases, see Fig. 1. In our model, we track the colony dynamics (*N*, *P*, and *V*, as well as the spatiotemporal distribution of foragers and resources) during the growth phase, then use the colony state at the end of that phase to determine the reproductive output of the colony (number of new queens). We then assume constant mortality and success factors for the hibernation and establishment phases, respectively, to determine the number of colonies *R* present during the next growth phase. This approach allows us to model the reproductive, hibernation, and establishment phases as a single step. Mathematically, then, the model has two phases that repeat each year: a continuous-time colony growth phase, followed by a discrete-time jump for the reproduction-hibernation-establishment phase.

Below, we provide a complete description of the model. In Sect. 2.2, we give a detailed description of the DEB state variables. Section 2.3 describes the mathematical expressions for each of the energy fluxes involved in the vital functions of the colony. In Sect. 2.4 we describe the application of the Maximum Entropy Principle to compute the spatial distribution of foragers of both types. Finally, in Sect. 2.5 we give the differential equations and update rules that describe the dynamics of the state variables *N*, *P*, *V*, *R* and the landscape variables $(F_i^N)$ and $(F_i^P)$.

### Energy Budget Variables

**Fig. 2:**
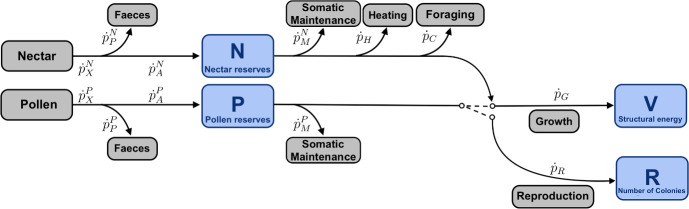
Diagram of the DEB model for a wild bumble bee colony. The switch represents a metabolic threshold at the end of the season. State variables are shown in blue and energy fluxes in gray. Their meanings are described in Sections 2.2 and 2.3 respectively (Color figure online)

Below, we specify what exactly each of the four energy budget variables represents. The equations governing their time-dependence are given in Section 2.5. Figure 2 schematically summarizes the DEB model and represents the connections between all state variables and energy fluxes.

**Nectar reserves **(*N*): This variable refers to the nectar stored in the nest. Nectar (which has a high concentration of sugar) is the main source of energy for the colony. Note that this state variable does not include the nectar in the landscape ($F_i^N$).

**Pollen reserves **(*P*): This variable refers to the pollen stored in the nest. The pollen (which is largely composed of proteins) is the main source of amino acids used for body development. Pollen reserves are critical for maturation of the brood. As with the nectar, this state variable does not include the pollen in the landscape ($F_i^P$).

**Structural energy **(*V*): This variable refers to the energy stored in the bodies of all foraging bees and brood. In particular, it is used as a proxy for the total numbers of foragers and brood, in the sense that both are assumed to be proportional to *V*; see ‘somatic maintenance’ in Section 2.3.

**Number of colonies **(*R*): This variable refers to the number of independent colonies (i.e., the number of queens) in the landscape.

For computational tractability, we assume that all of the nest sites are concentrated in a single location. This assumption is justifiable in the present context (see Sect. 4) but can be relaxed - to do so, we would need to define and track a separate copy of the state variables and forager distributions for each nest.

Variables *N*, *P*, and *V*, evolve continuously through differential equations from the moment in which the first worker bees emerge until they all die. Variable *R* evolves through a reproductive impulse that occurs from the moment all worker bees die until the new worker bees emerge. In our simulations, the continuous evolution normally accounts for a total of 150 days computed numerically in time steps of 0.1 days, whereas the reproductive impulse accounts for the remainder of the year in a single step.

### Energy Fluxes

A basic step in the construction of a DEB model is the definition of the energy fluxes, which are standardized (van der Meer 2006; Sousa et al. 2008). The energy fluxes for our model are illustrated in Fig. 2 and defined below.

**Ingestion (nectar **$\dot{p}^N_X$** and pollen** $\dot{p}^P_X$): We take “ingestion" to mean the floral resources collected from the landscape. Mathematically, we define: 
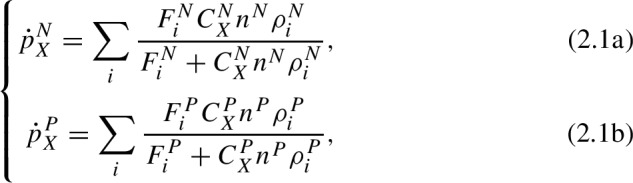
 where $F_i^N$ and $F_i^P$ are the amount of pollen and nectar resources in landscape patch *i*, $C_X^{N}$ and $C_X^{P}$ are the nectar/pollen collection rates per foraging bee, $n^N$ and $n^P$ are the number of nectar and pollen foragers, $\rho _i^N$ and $\rho _i^P$ are the probability density of nectar and pollen foragers in patch *i* (see (2.10)). Equations (2.1) mean that ingestion is proportional to landscape resource availability when the latter is limiting (e.g., $F_i^N < C^N_X n^N \rho ^N_i$, or similarly with *P* in place of *N*), and proportional to forager density when the landscape resources are abundant (e.g. $F_i^N > C^N_X n^N \rho ^N_i$, sim. for *P*). In other words, increasing the number of foragers only increases ingestion if there is resource available in the landscape, and increasing the landscape resources only increases ingestion if there are additional foragers available to collect it.

**Faeces (nectar **$\dot{p}^N_P$** and pollen** $\dot{p}^P_P$): These variables represent the quantity of ingested resource that can not be incorporated into the colony reserves. Mathematically, we define: 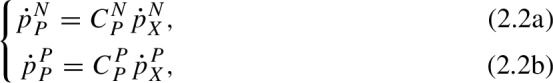 where $C^N_P$ and $C^P_P$ are constants of proportionality that dictate what proportion of the ingested food will not be incorporated into the reserves of the colony.

**Assimilation (nectar **$\dot{p}^N_A$** and pollen** $\dot{p}^P_A$): These variables represent the quantity of resource ingested that is actually incorporated into the colony reserves. Mathematically, we define: 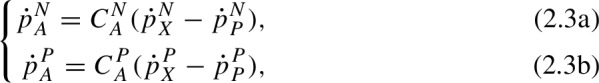 where $C^N_A$ and $C^P_A$ are proportionality constants that dictate what proportion of the incorporated food will actually be digested.

Note that, for simplicity, in simulations we will assume that the resource assimilation is perfect, i.e., $C_N^A=C_P^A=1$ and the resources lost in the form of faeces is small, i.e., $C_N^P = C_P^P=0$.

**Somatic maintenance (nectar **$\dot{p}^N_M$** and pollen **$\dot{p}^P_M$): This quantity is the turnover and maintenance of structural mass, summed over all individuals in the colony. Mathematically, we define: 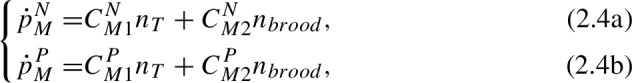 with $n_T$ being the total number of foragers ($n_T=n^N+n^P$) and $n_{brood}$ the total brood population. $C^N_{M1}$ and $C^P_{M1}$ are proportionality constants for somatic maintenance of foragers, and $C^N_{M2}$ and $C^P_{M2}$ are proportionality constants for somatic maintenance of brood. To maintain the simplicity of the model we will assume that the total number of foragers and brood are both proportional to the structural energy: 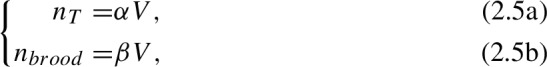 for some positive constants $\alpha ,\beta$.

**Heating **($\dot{p}_H$): In a classic DEB framework, heating refers to the energy spent to maintain an individual’s body temperature. At a colony level, these individual energy costs are summed and, in addition, there is energy spent heating the nest (nest temperature is fundamental for brood development) (Gradisek et al. 2023). Heating energy is derived from nectar reserves. Mathematically, we define:

2.6 $$\begin{aligned} \dot{p}_H = C_H n_{\max }, \end{aligned}$$

where $C_H$ is a proportionality constant and $n_{\max }$ is the maximum size attained by the colony over the course of the season, which we assume to be proportional to the size of the nest. Heating may also depend on environmental temperature. We do not consider this factor here, but, in future work, dependence on environmental temperature could be included through the addition of appropriate terms in (2.6).

**Foraging **($\dot{p}_C$): Foraging energy is the energy spent in movement during foraging. It is related to the foraging range of the colony (see Sect. 2.4 for details on the forager distribution), with bees travelling to nearby foraging locations consuming less energy than those travelling further away. Foraging energy is obtained through nectar reserves. Mathematically, we define:

2.7 $$\begin{aligned} \dot{p}_C = C_C\sum _i (n^N \rho ^N_i+n^P \rho ^P_i) d_i, \end{aligned}$$

where $C_C$ is a proportionality constant, $\rho _i^N,\rho _i^P$ are, respectively, nectar and pollen forager density (see equation (2.10)), and $d_i$ is the distance from the nest to resource patch *i*.

**Growth **($\dot{p}_G$): Colony growth refers to the increase in structural energy (*V*) as a result of nectar (*N*) and pollen (*P*) resource consumption. In the classic DEB framework, an increase in *V* reflects an increase in the size of individuals. At a colony level, it refers to the increase in the number of individuals in the colony. Brood is known to consume mostly pollen, so growth energy is derived from pollen reserves. Mathematically, we define:

2.8 $$\begin{aligned} \dot{p}_G = \kappa \frac{P}{P+\sqrt{V}\epsilon _P} \, \frac{N}{N+\sqrt{V}\epsilon _N}, \end{aligned}$$

where $\sqrt{V} \epsilon _N$ and $\sqrt{V} \epsilon _P$ are, respectively, the minimal amount of nectar and pollen resources necessary for growth. In our work here, we do not distinguish between wild and crop flower pollen and nectar, though such a distinction could be made if appropriate. Conversion of pollen to structural energy occurs at rate $\kappa$, which is a standard notation in the dynamic energy budget literature.

**Reproduction **($\dot{p}_R$): This flux is the energy allocated to reproduction. In standard DEB models for individuals, this flux would include maturity and maintenance thereof, as time to maturation is generally significant. The reproductive potential of a bumble bee colony, however, is chiefly a function of the number of worker bees at the end of the growth phase (Fig. 1). In our DEB framework then, the energy required for reproduction is a function of the structural energy stored in the colony, and does not include a maturation flux. Mathematically, we define:

2.9 $$\begin{aligned} \dot{p}_R = C_R \, R \,\tan ^{-1}\left( \left( \frac{V}{R}-\epsilon _R\right) \,\psi \right) , \end{aligned}$$

where $C_R$ is a proportionality constant that indicates how many new queens a colony can produce, *R* is the reproductive outcome ( number of colonies), *V* is the structural energy of the colony ( number of foragers and brood) and $\epsilon _R$ is the minimum structural energy of the colony (i.e., minimum colony size) required for reproduction. Note that colony reproduction is defined using a $\tan ^{-1}$ function, which is maximal (equal to $C_R$) when the structural energy of each colony is large ($\frac{V}{R} > \epsilon _R$), zero when the structural energy is small ($\frac{V}{R} < \epsilon _R$), and increases linearly with the structural energy of the colony when $\frac{V}{R} \approx \epsilon _R$. The parameter $\psi$ is a small positive constant that controls the slope of the $\tan ^{-1}$ function, that is, the rate at which queen production increases with *V*.

### Forager Distribution

To compute the time evolution of the state variables (eqs. (2.10)-(2.13)) we first need to find the nectar and pollen forager spatial distribution ($\rho ^N_i$ and $\rho ^P_i$), where *i* indexes patches, in a given landscape with nectar and pollen resources ($F^N_i,F^P_i$), for a given bee population ($n^N, n^P$). Finding the spatial distribution of populations is a common problem in spatial ecology, and many methods have been developed for this purpose (Austin 2007; Elith and Leathwick 2009). Here, we use the Maximum Entropy principle (MaxEnt), which has been extensively used in ecology (O’Dwyer et al. 2017; Phillips et al. 2006), and recently to the particular case of finding forager spatial distributions (Capera-Aragones et al. 2023), that we summarize below.

The application of the MaxEnt principle to a particular case, i.e., the case in which the energy function is assumed to not depend on the forager distribution, leads to the *Boltzmann Distribution* (Capera-Aragones et al. 2023): 
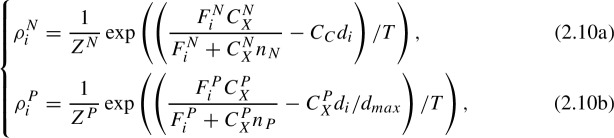


A consequence of these definitions (2.10), is that pollen foragers may go as far as $d_{max}$ if such travel is necessary to fill the colony pollen requirements, even at a significantly increased foraging cost ($\dot{p}_C$). However, the distance traveled by nectar foragers is limited by the energy returns, i.e., the energy gained from a full nectar load must be at least as large as the energy spent in travelling to the resource patch. In (2.10) the term 1/*T* refers to the efficiency (optimality) of the foraging strategy, with $T \rightarrow 0$ corresponding to optimal distribution of foragers (or Ideal Free Distribution) and $T \rightarrow \infty$ corresponding to randomly distributed foragers (or uniform distribution). $Z^N$ and $Z^P$ are normalization factors given by

$$\begin{aligned} Z^N&= \sum _i \exp \left( \frac{1}{T}\left( \frac{F^N_iC_X^N }{F^N_i+C_X^N n_N}-C_C d_i\right) \right) , \\ Z^P&= \sum _i \exp \left( \frac{1}{T}\left( \frac{F^P_iC_X^P }{F^P_i+C_X^P n_P}-C^P_X d_i/d_{max}\right) \right) . \end{aligned}$$

In order to find the distribution of nectar and pollen foragers, we first need to know the total number of nectar and pollen foragers ($n^P$ and $n^N$). These quantities are given by 
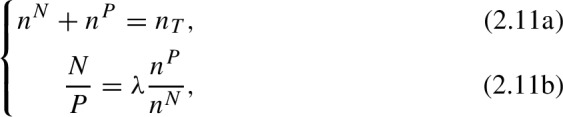
 where $\lambda$ is a parameter that determines the proportion of nectar foragers to pollen foragers in a landscape with unlimited floral resources, based on the nectar and pollen reserves in the colony, and $n_T=\alpha V$ for some constant $\alpha$. After rearranging terms in the previous equations, we obtain the number of nectar foragers ($n^N$) and pollen foragers ($n^P$) as a function of the total number of foragers ($n_T$), the nectar reserves (*N*) and the pollen reserves (*P*), 
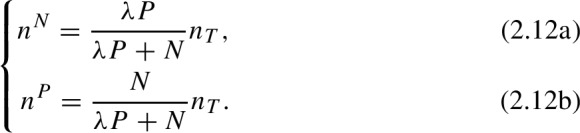


To make the model numerically stable, we add small correction terms to (2.12) to account for the cases in which $N\approx 0$ and $P\approx 0$. The final equations are 
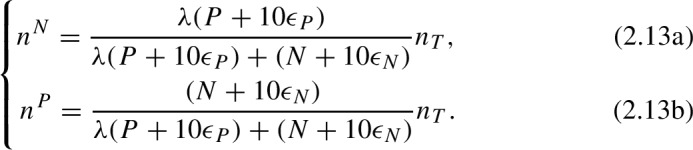
 With this correction, when the colony reserves (*N* or *P*) approach zero, the relative proportion of pollen and nectar foragers are determined by the minimal pollen and nectar needed for growth ($\epsilon _N$ and $\epsilon _P$). Equations 2.10 and 2.13 together allow us to determine the spatial distribution of foragers.

### Dynamics of the Model

Having defined the energy fluxes in the model, we now use these to develop the dynamical system for the time evolution of the bumble bee colonies (singular in year 1 and possibly multiple in later years). A summary of the model variables is given in Table 1.

The overall process is as follows. Letting *L* denote the length of the growth phase in days, successive growing seasons correspond to time intervals [0, *L*), [*L*, 2*L*), so, for example, the $30^{\text {th}}$ day of the $3^{\text {rd}}$ growing season corresponds to $t=2L+30$. During each growth phase, i.e., for $t\in [kL,(k+1)L)$ for integer $k\ge 0$, three of the energy budget variables *N*, *P*, *V*, and the resource values $(F_i^N),(F_i^P)$ are updated according to differential equations, given below, while the colony number *R* does not change. Note that the forager distributions $(\rho _i^N),(\rho _i^P)$ are computed in real-time from the values of the other state variables, according to equations (2.10). At the end of each growth phase, i.e., for $t \in \{L,2\,L,\dots \}$, the values $N(t^-):= \lim _{s\rightarrow t^-} N(s)$, $P(t^-):= \lim _{s\rightarrow t^-} P(s)$, and $V(t^-):= \epsilon _V$, immediately preceding the reproductive phase are used to update the values of the colony number *R*(*t*) and, subsequently, the structural volume *V*(*t*), that are used to begin the new growth phase. The values of *N*, *P* are reset to 0 at the start of each growth phase. The amount of resources in the landscape at the beginning of each growth phase is reset according to the scenario being studied.

**Table 1:** Summary of variables, their meaning, and the equations in which they appear

**Symbol**	**Meaning**	**Equations**
*N*	Nectar storage by all colonies	(2.14a)
*P*	Pollen storage by all colonies	(2.14b)
*V*	Structural energy (# of bees and brood) of all colonies	(2.14c)
*R*	Number of colonies	(2.14d)
$\rho _i^N$	Probability density of nectar foragers in patch *i*	(2.10a)
$\rho _i^P$	Probability density of pollen foragers in patch *i*	(2.10b)
$F _i^N$	Nectar in patch *i* of the landscape	(2.15a)
$F _i^P$	Pollen in patch *i* of the landscape	(2.15b)

**Colony growth phase:** Once the queen has selected a nest site and her first set of worker bees have matured and are foraging, the colony growth phase begins. During this time, i.e., for $t\in [kL,(k+1)L)$ and integer $K\ge 0$, the dynamic variables *N*, *P*, *V*, *R* and $(F_i^N),(F_i^P)$ evolve as follows. First, the energy budget variables:

2.14a $$\begin{aligned} \frac{dN}{dt}&= \dot{p}^N_A -\dot{p}^N_M - \dot{p}_H - \dot{p}_C- \theta N, \end{aligned}$$

2.14b $$\begin{aligned} \frac{dP}{dt}&= \dot{p}^P_A -\dot{p}^P_M -\dot{p}_G, \end{aligned}$$

2.14c $$\begin{aligned} \frac{dV}{dt}&= \frac{R}{E_G}\dot{p}_G - (\delta _0 +\delta _Ne^{-N/\epsilon ^N}) V, \end{aligned}$$

2.14d $$\begin{aligned} \frac{dR}{dt}&= 0, \end{aligned}$$

where the energy fluxes are described and formulated in Sect. 2.3. The remaining terms are as follows: The term $-\theta N$ represents loss of nectar due to evaporation. $E_G$ is a measure of the proportion of pollen that will transform into structural energy. This parameter is related to the rate at which queens lay eggs. The parameter $\delta _0$ is the ‘‘natural’’ death rate and $\delta _N e^{-N/\epsilon ^N}$ is an additional death rate due to low nectar resources.

We also track the floral resources in the landscape, via the following equations:

2.15a $$\begin{aligned}&\frac{dF^N _i}{dt}= F^N_{0i} - \dot{p}_X^N-\gamma ^N\, (F^N _i)^2, \end{aligned}$$

2.15b $$\begin{aligned}&\frac{dF^P _i}{dt}= F^P_{0i} - \dot{p}_X^P -\gamma ^P (F^P _i)^2, \end{aligned}$$

where the the first term ($F^{P/N}_{0i}$) represents the production of nectar or pollen in patch *i* (which is related to the number of flowers in the region and is generally heterogeneous and dynamic), and the second term represents the consumption of nectar or pollen by foragers. The third term is phenomenological and is included to ensure that the available floral resources do not grow without bound but, instead, saturate at some maximal level.

**Reproduction, Hibernation, and Establishment phases:** In spring, when the foraging season is about to start, the number of colonies (*R*) is determined by the size of the colonies from the previous season, with factors accounting for queens that perish during the winter. We assume 100% colony establishment success. In addition, the colony pollen and nectar reserves (*P* and *N*) are set to zero, and the structural energy (*V*) is set to a small value which accounts only for the presence of queen bees. Mathematically, for $t \in \{L,2L,\dots \}$:

2.16a $$\begin{aligned} N(t)&= P(t) = 0, \end{aligned}$$

2.16b $$\begin{aligned} R(t)&= R(t^-) + \dot{p}_R - \delta _R R, \end{aligned}$$

2.16c $$\begin{aligned} V(t)&= \epsilon _VR(t) \end{aligned}$$

where $\dot{p}_R$ is colony reproduction (production of queens) in the fall, $\delta _R R$ is queen death during the winter, $\epsilon _VR$ is the structural energy corresponding to all queen bees.

### Parameter Values

All of the default parameter values used in our computer simulations are specified in Table 2. Some parameter values are taken from empirical studies, while others are determined qualitatively by requiring the model to correctly reproduce certain expected behaviours following established methods (Carturan Bruno et al. 2023; Kravtsova et al. 2023; Tyson and Novak 2020).

**Table 2:** Summary of the parameters, their meaning, the equations in which they appear, their values used in our simulations, their units, and the reference s used for their approximation. References cited as ‘Banks (2020)*’ (with the asterisk) indicates that the value is based on that found in the mentioned source but adapted to the specifications of our model. All parameters referred as ‘Assumption’ are explained in the SI

**Symbol**	**Meaning**	**Equations**	**Value**	**Units**	**Reference**
$C^{P}_X$	Nectar collection rate	(2.1)	1.2	(ml)/(#Days)	Banks (2020)*
$C^{N}_X$	Pollen collection rate	(2.1)	0.8	(g)/(#Days)	Banks (2020)*
$C^{N/P}_P$	Nectar/pollen to faeces rate	(2.2)	0	-	Assumption
$C^{N/P}_A$	Nectar/pollen assimilation rate	(2.3)	1	-	Assumption
$C^{N}_{M1}$	Forager nectar consumption rate	(2.4)	0.025	(ml)/(#Days)	Banks (2020)*
$C^{P}_{M1}$	Forager pollen consumption rate	(2.4)	0.04	(g)/(#Days)	Banks (2020)*
$C^{N}_{M2}$	Brood nectar consumption rate	(2.4)	0	-	Assumption
$C^{P}_{M2}$	Brood pollen consumption rate	(2.4)	0.01	(g)/(#Days)	Banks (2020)*
$C_H$	Nectar consumption for heating rate	(2.6)	0.03	(ml)/(#Days)	Banks (2020)*
$C_C$	Nectar consumption for travelling rate	(7)	0.4	(ml)/(#DaysKm)	Banks (2020)*
$\theta$	Nectar evaporation rate	(2.14a)	0.01	1/Days	Assumption
$\kappa$	Conversion rate of pollen to structural energy	(2.8)	1	1/Days	Assumption
$\epsilon _N$	Minimal quantity of nectar for growth	(2.8)	0.1	ml	Assumption
$\epsilon _P$	Minimal quantity of pollen for growth	(2.8)	0.5	ml	Assumption
$\delta _0$	Forager death rate	(2.14c)	0.05	1/Days	Banks (2020)
$\delta _N$	Nectar-lacking death rate	(2.14c)	1	1/Days	Assumption
$E_G$	1/P required to increase V rate	(2.14c)	1/8.5	g/#	Banks (2020)
$\lambda$	Proportion of pollen to nectar stored	(2.11),(2.12),(2.13)	5	-	Free (1955)
$\alpha$	Proportion of # of bees to structural energy	(2.5)	1	-	Assumption
$\beta$	Proportion of # of brood to structural energy	(2.5)	4	-	Banks (2020)
*T*	Inverse efficiency (optimally) of forager strategy	(2.10)	0.1	-	Assumption
$\gamma ^N$	Landscape nectar saturating rate	(2.15a)	0.001	1/ml	Assumption
$\gamma ^P$	Landscape pollen saturating rate	(2.15b)	0.001	1/ml	Assumption
$C_R$	Maximum # of new queens	(2.9)	5	1/Days	Assumption
$\delta _R$	Queen’s death rate during reproductive impulse	(2.16a)	0.12	1/[Impulse time]	M A Becher et al. (2018)
$\epsilon _V$	Value of V to start colony growth phase	(2.16a)	0.001	#	Assumption
$\epsilon _R$	Size of the colony necessary for reproduction	(2.16a)	100	#	Assumption
$\psi$	Tolerance for reproduction when $V/R < \epsilon _R$	(2.9)	0.2	-	Assumption

### Model Implementation

We simulated the model in Python, using Euler’s method to solve the ODEs. See the Code Statement for details on how to access the code.

**Fig. 3:**
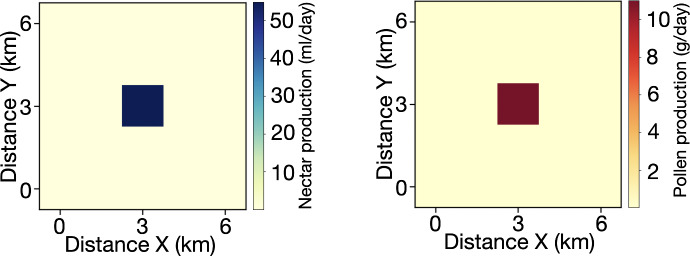
Example of the spatial distribution of nectar (left) and pollen (right) resource in the landscape. The colony is located at the center, where there is also a high density patch of pollen and nectar (dark green (left) and red (right)). A low quantity ’background’ of flowers is spread uniformly throughout the remainder of the landscape (pale yellow) (Color figure online)

The simulated landscape is a square domain with dimensions 6 km by 6 km. The landscape is internally structured as a square spatial grid with spacing equal to 1.2 km (except for the simulations in section 3.1.3, where the resolution is set to 0.67 km). Flower patches are assumed to exist at each node of the lattice, and each may contain pollen, nectar, both resources, or none. A sample landscape is shown in Fig. 3. The nesting site is always located at the geographical centre. The bee foraging range is set to $\approx 3$ km for nectar foragers, but is arbitrarily large for pollen foragers. This difference is due to foragers requiring nectar as an energy source for travelling, but not pollen. In the following sections, we explain the particular landscape considered in each scenario. Simulations were run over just the growth phase ($L=150$ days), or over multiple years, with a growth phase in each year of *L* days. Time steps are set to 0.1 days.

## Results

We divide our results into three parts: First, we use the model to predict, over a single season, colony population dynamics^1^ and the spatial distribution of foragers in landscapes with different spatio-temporal patterns of floral resource, including only background flowers and crop flowers (i.e., no wildflower enhancements). This work provides us with a baseline understanding of colony growth in the absence of wildflower enhancements.

Second, we use the model to predict colony population dynamics and the evolution of the number of colonies, across multiple years, in landscapes with varying resource density, but still without wildflower enhancements.

Third, we use the model to predict how crop pollination services depend on the characteristics of wildflower patch enhancements. We aim to determine the best location, composition (proportion of pollen and nectar), quantity (density of floral resources), and bloom time for the wildflowers in terms of the benefit to crop pollination services.

###  Colony Response to Resource Limitation, Agricultural Intensity, and Foraging Distance

#### Nectar- and Pollen-Limited Landscapes Differently Affect Colony Structural Energy

We investigate colony growth in a landscape with (a) abundant pollen and nectar resources, (b) abundant pollen but limiting nectar resources, and (c) abundant nectar but limiting pollen resources. The landscape consists of a wildflower patch with a high quantity of pollen and nectar co-located with the nest site at the centre of the domain, and a uniform low density ’background’ of pollen and nectar throughout the remaining landscape (see Fig. 3). In scenario (b), the quantity of nectar in the centre patch is reduced by a factor of 10 with respect to Fig. 3 (left), while in scenario (c), the quantity of pollen in the center patch is reduced by a factor of 10 with respect to Fig. 3 (right).

**Fig. 4:**
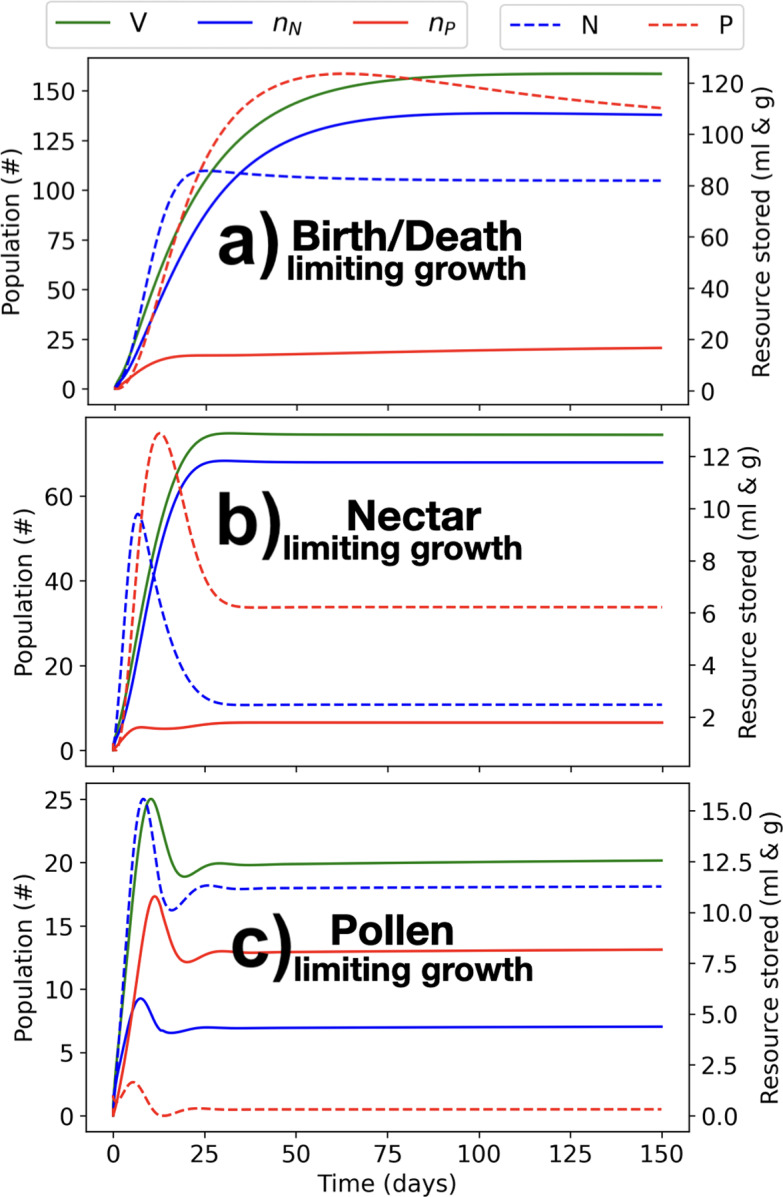
Evolution of the structural energy of the colony ($V=n_N+n_P$), nectar foragers ($n_N$), pollen foragers ($n_P$) (left vertical axis), nectar reserves (*N*) and pollen reserves (*P*) (right vertical axis). In (a), pollen and nectar are abundant and available; in (b) pollen is abundant, but nectar is not; in (c) nectar is abundant, but pollen is not. The simulated landscape consist s of low-quantity ‘background’ flowers with constant uniform resource production across all nodes of the simulated grid, with 0.1 *g*/*day* production of nectar and 0.1 *ml*/*day* production of pollen at each node of the simulated grid, and a nectar and pollen patch located at the central node which produces 500 *ml*/*day* of nectar and 100 *g*/*day* of pollen (top), 50 *ml*/*day* of nectar and 100 *g*/*day* of pollen (centre), and 500 *ml*/*day* of nectar and 10 $g/Km \cdot day$ of pollen (bottom) (color figure online)

Fig. 4 shows how colony size reaches a characteristic maximum size in all three cases (a,b,c). However, the causal mechanism is different in each case: In (a), we see that colony reserves in both pollen and nectar are high throughout, but the colony stops growing because there is a maximum rate at which the queen can lay eggs, while the death rate of worker bees increases with colony size. In (b), we see that the nectar reserves are small, which limits the growth of the brood and increases the mortality of worker bees according to Eq. 2.8, overall leaving the nest with a smaller stationary size than in (a). In (c), we see that the pollen reserves are small, which limits the growth of the colony according to Eq. 2.8.

**Fig. 5:**
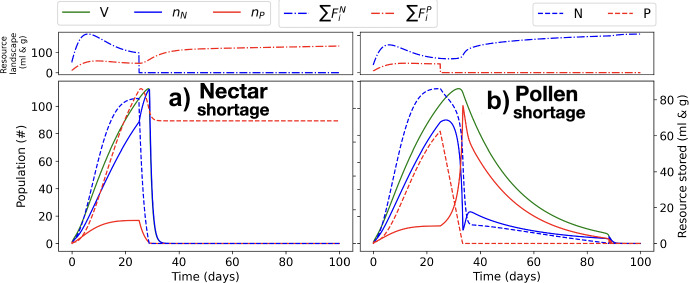
Colony response to a sudden and persistent drop in landscape resources. A shortage in nectar (left) or pollen (right) occurs at day 25. The simulated landscape consists of constant low-quantity ‘background’ flowers with constant uniform resource production at 0.1 *g*/*day* of pollen and 0.1 *ml*/*day* of nectar at each node of the simulated grid, and a nectar and pollen patch located at the central node, which produces 500 *ml*/*day* of nectar and 100 *g*/*day* of pollen during the first 25 days and no nectar (left) or pollen (right) afterwards. Bottom frame in each subplot: Evolution of the structural energy of the colony ($V=n_N+n_P$), nectar foragers ($n_N$), pollen foragers ($n_P$) (left vertical axis), nectar reserves (*N*) and pollen reserves (*P*) (right vertical axis). Top frame in each subplot: Evolution of the nectar ($\sum F_i^N$) and pollen ($\sum P_i^N$) resources on the landscape (color figure online)

We investigate the effect of a nectar/pollen shortage on colony growth and survival. We simulate the landscape in Fig. 3 for 25 days, and we remove all nectar/pollen resources in the landscape after day 25. We then observe colony evolution during the following 75 days. Figure 5 shows how population size evolves before and after the induced resource shortage. When we induce a nectar shortage (a), we see a fast decay of nectar reserves (within around 2 days), and following the emptying of nectar reserves, an increased mortality of foraging bees that drives the colony to extinction (through an exponential decay, after only 5-10 days from the nectar shortage). When we induce a pollen shortage (b), we see pollen decaying at a smaller rate than nectar in (a) due to its low volatility and lower consumption respect to nectar at the high traveling costs associated to a colony that misses one type of resource. In (b), the colony size not only does not shrink but even increases during a short time after the pollen shortage, the time until pollen reserves are completely depleted (after around 5-10 days from pollen shortage). Following the depletion of pollen reserves, colony growth stops and foragers begin to search mostly for pollen, which slowly reduces colony size (linear decay), nectar reserves, and ultimately drives the colony to extinction about 50-70 days from the onset of pollen shortage.

#### Heating Costs can Lead to Extinction After Early Mass Crop Bloom

We investigate the effects that a mass-flowering crop (MFC) can have on colony survival as a function of the background level of flowers in the landscape, under the assumption that the energy cost for heating the nest is proportional to the nest maximal size. We simulate the landscape depicted in Fig. 3, in which the center patch represents the crop and the surrounding area represents the background flowers. The crop is set to bloom only between day 65 and 85. Simulations are run for 150 days.

**Fig. 6:**
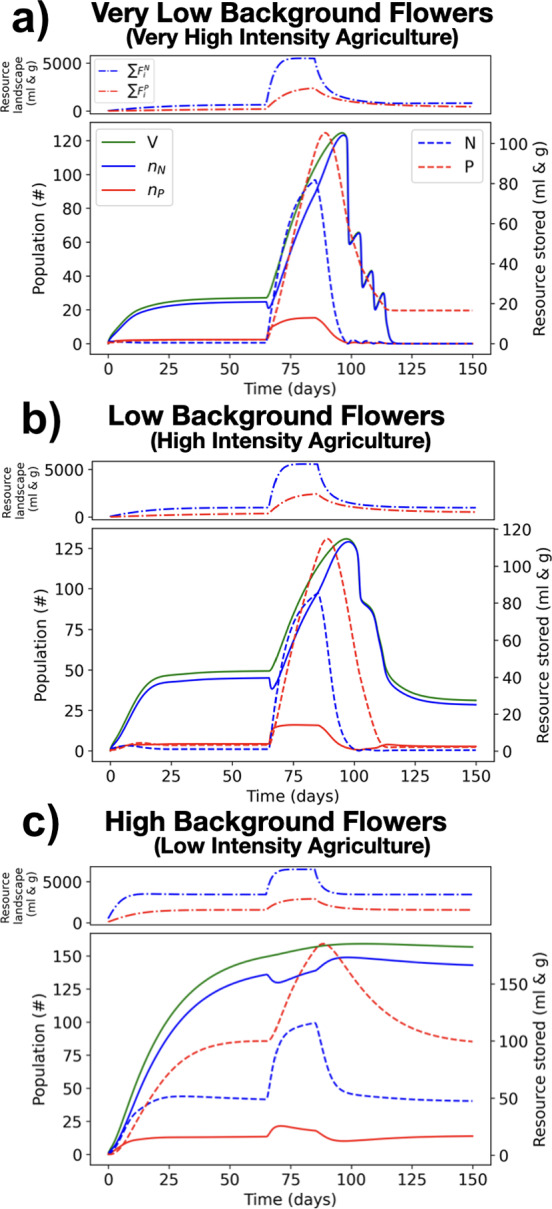
Colony response to the temporary bloom of a mass flowering crop. The crop patch is centered at the nest, and bloom occurs between days 65 and 85. Crop floral production is 100 g/day for pollen and 500 ml/day for nectar. The simulated landscape consists of a constant uniform production of pollen and nectar across the 5x5 grid, at three different levels: (a) 0.4 g/ day of pollen and 10 ml/day of nectar, (b) 0.8 g/day of pollen and 20 ml/day of nectar, and (c) 2 g/day of pollen and 50 ml/day of nectar. Bottom frame in each subplot: Time evolution of the structural energy of the colony ($V=n_N+n_P$), nectar foragers ($n_N$), pollen foragers ($n_P$) (left vertical axis), nectar reserves (*N*) and pollen reserves (*P*) (right vertical axis). Top frame in each subplot: time evolution of the nectar ($\sum F_i^N$) and pollen ($\sum P_i^N$) resources on the landscape (color figure online)

Fig. 6 shows how population size evolves before and after the mass-flowering crop (MFC) bloom. When there is a very low quantity of background flowers (a), representing a landscape with intensive agriculture, where pesticides or mowing are applied to remove non-crop floral resources, the bee population is small before the crop bloom, increases quickly during crop bloom, and goes to extinction due to the increased heating costs associated to a larger colony after crop bloom. When background flowers are present in small quantities (b), the bee population is small, increases during crop bloom, and is reduced with respect to before the crop bloom, but does not go to extinction after crop bloom. When there is a large quantity of background flowers, the bee population is large (nearly maximum colony size), and the colony neither grows, nor shrinks, after crop bloom. According to Fig. 6, if the heating cost of a nest depends on the size of the nest, an MFC bloom in an intensively farmed landscape (where few floral resources other than the crop are available) can seriously threaten the survival of colonies. This result is consistent with field studies on bumble bees (Galpern et al. 2017; Kallioniemi 2017).

Heating cost exacerbates the burden of an overly large population, a phenomenon that can occur not only with massive crop blooms but in other scenarios in which a brief period of high nutrient levels is followed by a period of relative scarcity. It may be of interest for future studies to use the present modelling framework to investigate colony survival as a function of environmental temperatures, a feature that can easily be incorporated into the DEB framework developed here.

Fig. 6 suggests that MFC blooms present significant environmental challenges that require distinct adaptive responses from pollinator populations. While honey bees demonstrate resilience through behavioral adaptations such as cannibalism (Schmickl and Crailsheim 2001), bumble bee colonies face more pronounced difficulties due to their complex thermoregulatory requirements and resource management constraints. This differential adaptation capacity suggests potential biodiversity impacts near MFC blooms, as species-specific vulnerabilities influence population dynamics and community composition.

#### Growth of Structural Energy Varies with Minimum Foraging Distances for Pollen and Nectar

**Fig. 7:**
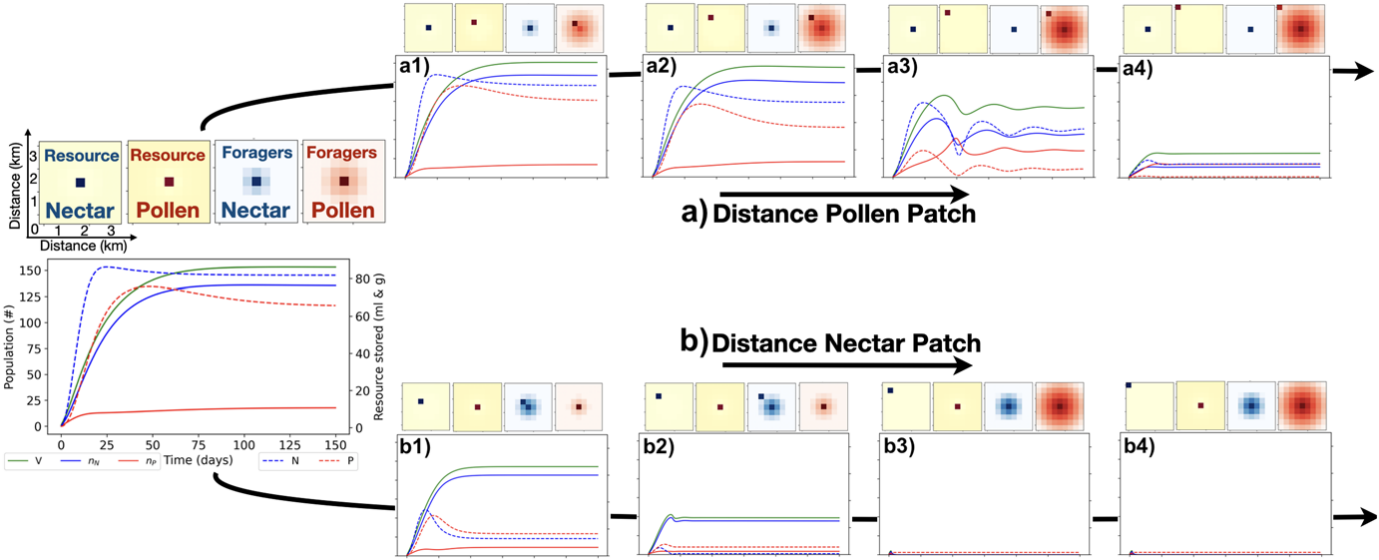
Colony response to increased foraging distances. The simulated landscape consists of constant low-quantity ‘background’ flowers with constant uniform resource production at 0.025 g/day for pollen and 0.025 ml/day for nectar in each of the nodes of the 10$\times$10 grid, as well as nectar patch producing 500 ml/ day and a pollen patch producing 100 g/day. The nectar and pollen patches are initially close to the nest and contiguous (left subplot), and then one or the other (a) for pollen and b) for nectar) is moved gradually further from the nest (series of subplots, top and bottom). Top frames in each subplot: Heat maps showing the spatial location and density of nectar and pollen resources on the landscape, and nectar and pollen foragers. Bottom frame in each subplot: Evolution of the structural energy of the colony ($V=n_N+n_P$), nectar foragers ($n_N$), pollen foragers ($n_P$) (left vertical axis), nectar reserves (*N*) and pollen reserves (*P*) (right vertical axis) (color figure online)

We investigate how the distance between the resource patches and the nest influences colony population dynamics. We simulate the landscape in Fig. 3, but with a resource patch (either pollen or nectar) that is progressively relocated farther from a central nest. Importantly, the spatial distribution of foragers is not assumed to be a fixed, normal distribution around the nest. Instead, our model allows the distribution to adapt to both the colony’s resource requirements and the costs associated with longer flights. Our simulation incorporates the energetic costs of travel; thus, foragers only extend their range when the net energy return (or resource gain) justifies the extra cost.

Fig. 7 (top) shows that, increasing the distance to the pollen patch broadens the spatial distribution of pollen foragers and raises travel costs, which in turn reduces colony size. In scenarios where the pollen patch is distant and the colony is moderately small, foragers are compelled to search beyond the immediate vicinity because nearby background flowers are insufficient to fulfill pollen demands. Conversely, Fig. 7 (bottom) shows that increasing the distance to the nectar patch has a more dramatic effect: not only does the colony shrink due to higher flight costs, but it eventually goes nearly extinction if the energy deficit becomes too severe.

It is critical to note that our findings do not suggest that the natural background of low-quantity flowers is inherently inadequate to sustain a bumble bee colony. Rather, they highlight that under conditions of increased patch-nest distance, the relative benefits of exploiting a targeted, high-yield resource become essential. When the colony is small and the energetic costs of long-distance foraging are prohibitive, foragers shift to exploiting the nearby background flowers, which can partially offset the loss of the patch resource.

###  Colony Number Saturates over Multiple Years

**Fig. 8:**
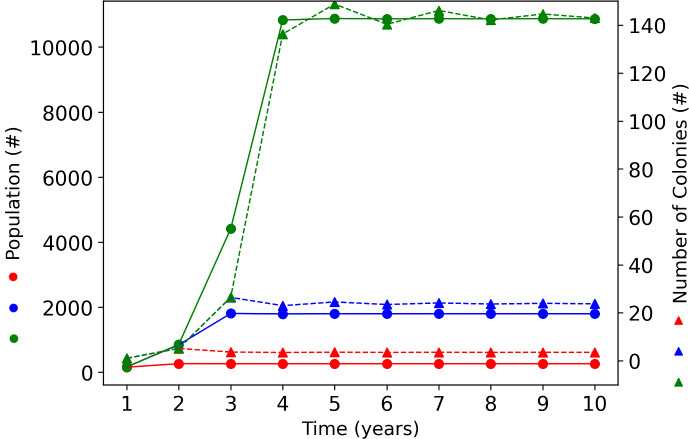
Colony response to constant resource levels over multiple years. The simulated landscape consists of a 5x5 grid with constant uniform resource production of pollen and nectar at each grid node at three different levels: (red) 10 *g*/*day* of pollen and 250 *ml*/*day* of nectar, (blue) 100 *g*/*day* of pollen and 2500 *ml*/*day* of nectar, and (green) 1000 *g*/*day* of pollen and 25000 *ml*/*day* of nectar. The colony metrics (foraging population $n_N+n_P$ (dots, left vertical axis) and number of colonies *R* (triangles, right vertical axis)) each year are sampled immediately before the reproductive impulse (Color figure online)

We simulate a landscape with constant and uniformly distributed pollen and nectar resource production over multiple years. Figure 8 shows the evolution of the structural energy (V) (which is proportional to the total population of foraging bees, $n_T=\alpha V$) and the number of colonies (R). We see how, starting in year 1 with a single colony, the number of colonies and foraging bees increases during the first years and saturates afterwards. The number of years to reach the saturation and the values of R and V at saturation depends on the quantity of resource in the landscape, with larger resource quantities allowing larger R and V at saturation and a longer time to reach saturation.

### Change in Crop Pollination Services Driven by Wildflower Enhancements

#### Change Within A Single Season: *Composition-compensatory* patches

In Capera-Aragones et al. (2024), the authors describe ‘Composition-compensatory’ wildflower patches as flower enhancements near crops which offer resources that are complementary to those provided by crop flowers (e.g., providing a pollen to nectar ratio that is different, or pollen of a different nutritional content). Capera-Aragones et al. (2022) found that such wildflower enhancements can increase crop pollination services, especially in intensively farmed landscapes, i.e., landscapes where the background level of wildflowers is low. However, Capera-Aragones et al. (2022) neglected to include the effects of population dynamics. With the model developed here we can explore these effects.

We define ‘Relative Composition’ as a measure of the proportion of nectar versus pollen produced by the crop relative to the wildflowers. Mathematically, we have:

3.17 $$\begin{aligned} \textit{Relative Composition} = 100 (\frac{F^N_{CROP}-F^P_{CROP}}{F^N_{CROP}+F^P_{CROP}})= 100 (\frac{F^P_{WF}-F^N_{WF}}{F^N_{WF}+F^P_{WF}}), \end{aligned}$$

where $F^N_{CROP}$ and $F^N_{CROP}$ are the nectar and pollen produced by the crop, and $F^N_{WF}$ and $F^N_{WF}$ are the nectar and pollen produced by the wildflowers. Relative Composition takes values in the range $(-100,100)\%$. As this metric is novel and perhaps not immediately intuitive, we give several examples. A value of $-100\%$ means the crop produces pollen but no nectar, while the wildflowers produce nectar but no pollen, and the exact opposite is true for a value of $100\%$. A value of $0\%$ means the crop and wildflowers each separately produce the same amount of pollen as they do nectar. A value of $20\%$ indicates that nectar production in the crop is 20% higher than the average of nectar and pollen production in the crop (e.g. take nectar, pollen production to be 3 and 2 respectively), while for wildflowers, pollen production is 20% higher than average. For a value of $-20\%$, the same holds except the roles of crop and wildflower are reversed.

We define the *Relative Change in Crop Pollination Services* as the change in crop pollination services upon addition of a wildflower (WF) patch. Mathematically, we have:

3.18 $$\begin{aligned}&\textit{Relative Change in Crop Pollination Services} =\end{aligned}$$

3.19 $$\begin{aligned}&\frac{(\textit{Pollination Services with WF}- \textit{Pollination Services without WF})}{ \textit{Pollination Services without WF}}. \end{aligned}$$

Similarly, we define the *Relative Quantity of Wildflowers* in a patch as the percentage relative to the quantity of crop flowers in the landscape:

3.20 $$\begin{aligned}&\textit{Relative Quantity WF} = \frac{(\textit{Quantity Crop Flowers}- \textit{Quantity WF})}{ \textit{Quantity Crop Flowers}}, \end{aligned}$$

and we define the *Relative Background Flowers* as the percentage of flowers not included in the crop or the wildflower patch relative to the total quantity of flowers in the landscape:

3.21 $$ \begin{aligned}&\textit{Relative Background Flowers} = \frac{(\textit{Total Flowers}- \textit{Flowers in Crop \& WF patch})}{ \textit{Total Flowers}}, \end{aligned}$$

The *Relative Background Flowers* is inversely proportional to the agricultural intensity in the farmed land.

**Fig. 9:**
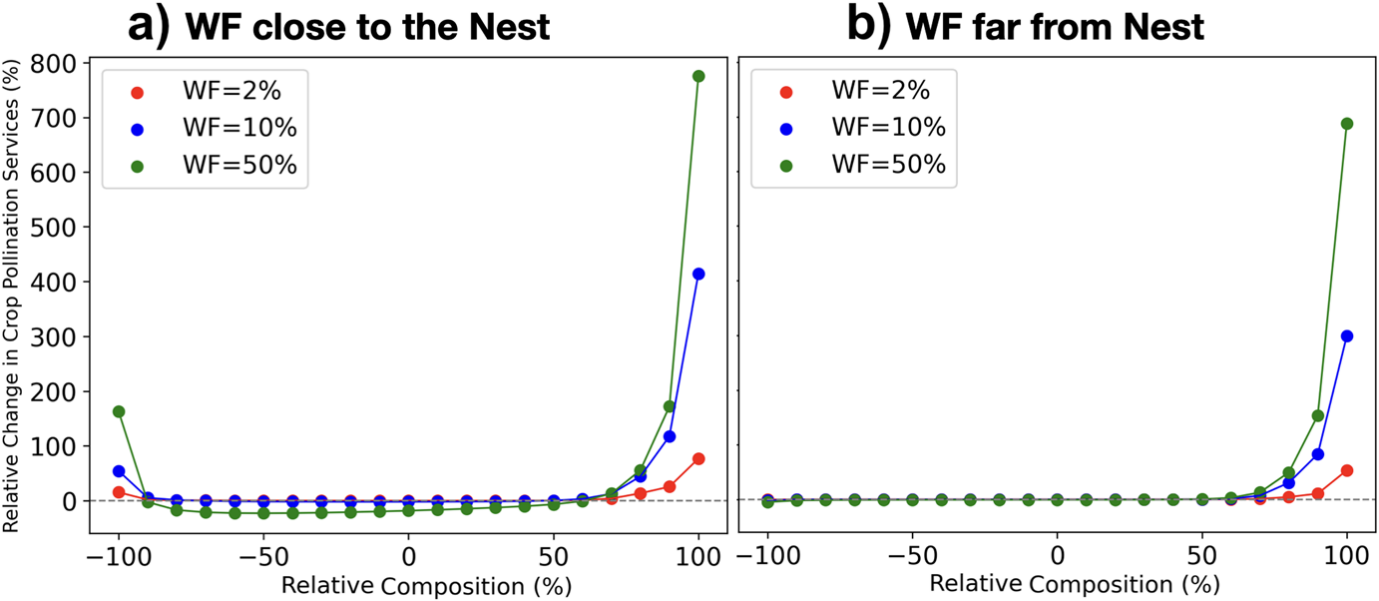
*Relative Change in Crop Pollination Services* as a function of the *Relative Composition* of the crop and the wildflower patch, for three different wildflower patch quantities (WF), representing 2% of the crop’s size (red lines), 10% (blue lines), and 50% (green lines). In a) the wildflowers and the crop are equi-distant from the nest (’WF close’). In b) the wildflowers are twice as far from the nest as the crop (’WF far’). The simulated landscape consist s of a constant uniform resource production of pollen at 0.1*g*/*day* and nectar at 0.1*ml*/*day* in each node of the 5x5 grid, a crop located on the right of the central node of the grid producing $\frac{|RC|}{100} \cdot 100$ g/day of pollen and $\frac{|1-RC|}{100} \cdot 500$ ml/day of nectar, and a wildflower patch located on the left of the central node of the grid producing $RWF\frac{|1-RC|}{100} \cdot 100$ g/day of pollen and $RWF\frac{|RC|}{100} \cdot 500$ ml/day of nectar. Where ’$\frac{RC}{100}$’ is the relative composition of pollen and nectar and ’*RWF*’ is the relative quantity of wildflowers respect crop flowers (Color figure online)

**Fig. 10:**
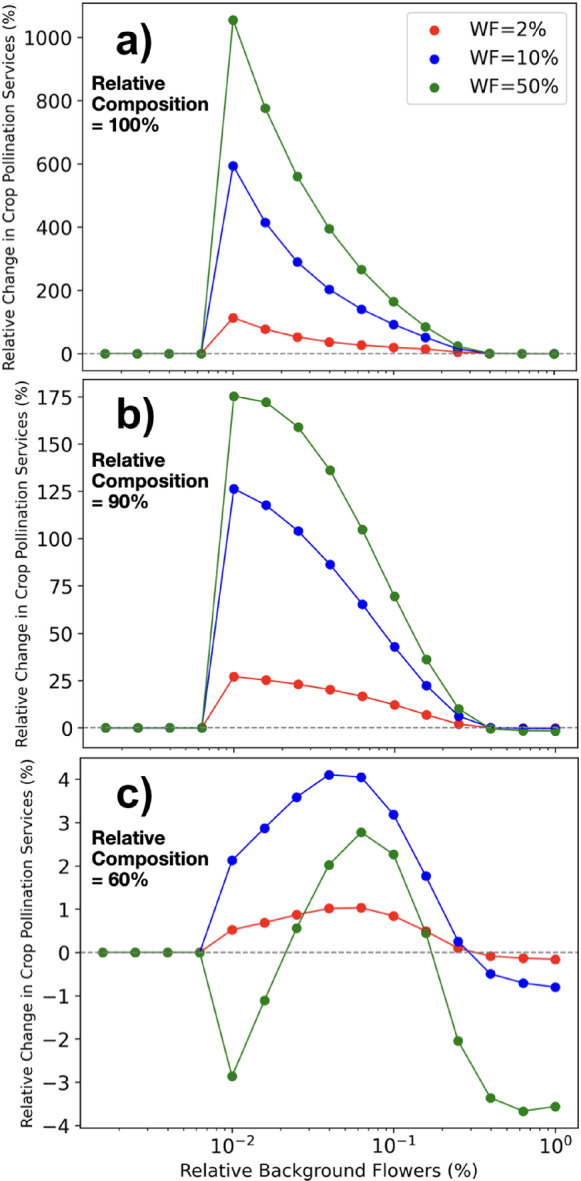
*Relative Change in Crop Pollination Services* as a function of the *Relative Background Flowers*, for three different wildflower patch quantities (WF) representing 2% (red lines), 10% (blue lines), and 50% (green lines) of the crop’s size, and three different *Relative Composition* levels, representing $100\%$ (top), $90\%$ (centre), and $60\%$ (bottom). The simulated landscape consists of a constant uniform resource production of pollen at 0.1g/day and nectar at 0.1 ml/ day in each node of the 5$\times$5 grid, a crop located on the right of the central node of the grid producing $\frac{|RC|}{100} \cdot 100$ g/day of pollen and $\frac{|1-RC|}{100} \cdot 500$ ml/day of nectar, and a wildflower patch located on the left of the central node of the grid producing $RWF\frac{|1-RC|}{100} \cdot 100$ g/day of pollen and $RWF\frac{|RC|}{100} \cdot 500$ ml/day of nectar. Where ‘$\frac{RC}{100}$’ is the relative composition of pollen and nectar and ‘*RWF*’ is the relative quantity of wildflowers with respect to crop flowers (Color figure online)

We investigate the *Relative Change in Crop Pollination Services* in a landscape containing four main ingredients: a crop, a wildflower patch (with variable quantity of flowers and location), a nest, and a background consisting of a small, uniformly distributed quantity of flowers. The crop and the wildflowers are set to bloom simultaneously between days 65 and 85, while the background flowers are set to bloom at all times. Figure 9 shows the *Relative Change in Crop Pollination Services* as a function of the *Relative Composition* for three different quantities of the wildflower patch and two different distances of the wildflower patch relative to the nest location. We see that as the *Relative Composition* increases, the *Relative Change in Crop Pollination Services* also increases if the crop is pollen deficient (i.e., a Relative Composition from 60% to 100%). But it does not increase (or increases less) when the crop is nectar deficient (i.e., a Relative Composition from -60% to -100%). In addition, we see that having a greater quantity of wildflowers is beneificial when the *Relative Composition* is high ($\approx -100$ or $\approx 100$), but is detrimental when is low ($\approx 0$), since then wildflowers can out-compete crop flowers for pollinators and drive a decrease in crop pollination services (the competition is especially evident when wildflowers are close to the nest).

Fig. 9 (a) shows that having the wildflowers close to the nest increases benefits when the *Relative Composition* is high, but increases drawbacks when it is low (especially when the quantity of wildflowers is high). This suggests that the location and quantity of the wildflower patch is critical when a high *Relative Composition* can not be achieved due to the composition of crop flowers. The results shown in Fig. 9, which are obtain ed using a different modelling approach than Capera-Aragones et al. (2022), are in agreement.

The *Relative Change in Crop Pollination Services* as a function of the *Relative Background Flowers* is shown in Fig. 10, for three different quantities of the wildflower patch (colour lines) and three different values of *Relative Change in Crop Pollination Services* (a,b,c panels). We see that the smaller the *Relative Background Flowers* (i.e., the more intense the agriculture), the higher is the increase in crop pollination services of adding wildflowers. This is true except when the *Relative Background Flowers* is too low, moment in which bee colonies go to extinction before the crop or wildflowers bloom, and pollination services are then zero. We also see that the benefits greatly increase with the *Relative Composition*, and that for low *Relative Composition*, adding large quantities of wildflowers may be detrimental due to competition.

#### Across Seasons Change: *Population-increase patches*

Blaauw Brett and Isaacs (2014) empirically showed that the addition of wildflowers patches adjacent to an agricultural crop can increase the pollination services of the crop over the course of a four years study. The wildflowers in Blaauw Brett and Isaacs (2014) were blooming all over the foraging season and the extension of the wildflower patch was of around 30% of crop’s size. Details of the relative composition of the wildflowers with respect to the crop, the exact quantity of wildflowers in the patch (size of the patch times density of flowers in the patch), the exact location of the wildflowers relative to the crop, and the intensity of the agriculture (background of flowers nearby the crop) are not directly specified in Blaauw Brett and Isaacs (2014), and can change the predictions of our model.

In this section, we want to find the characteristics of the wildflower patch that would most benefit crop pollination services. That is, we want to answer the five following questions:

1. Where to plant the wildflowers? (determine the location).
2. How many wildflowers? (determine the quantity and extent).
3. Which relative composition should the wildflowers have? (determine the type of wildflowers depending on their pollen/nectar composition).
4. When should the wildflowers bloom? (determine the bloom time).
5. Does the level of agricultural intensity change the results? (determine if changing the wildflower background causes qualitative changes on the predictions).

Note that maximizing crop pollination services does not equate to maximizing the well-being of colonies, because colonies benefit from resources other than the crop. From Fig. 10 we have already learned that the agricultural intensity does change how wildflower patches affect crop pollination services, but they do it in a predictable manner. In particular, we have seen that the crop pollination benefits of planting wildflowers increase monotonically with increasing agricultural intensity (or decreasing flower background). We therefore have an answer to question 5: the more intense the agriculture, the more vital the wildflower patches are.

**Fig. 11:**
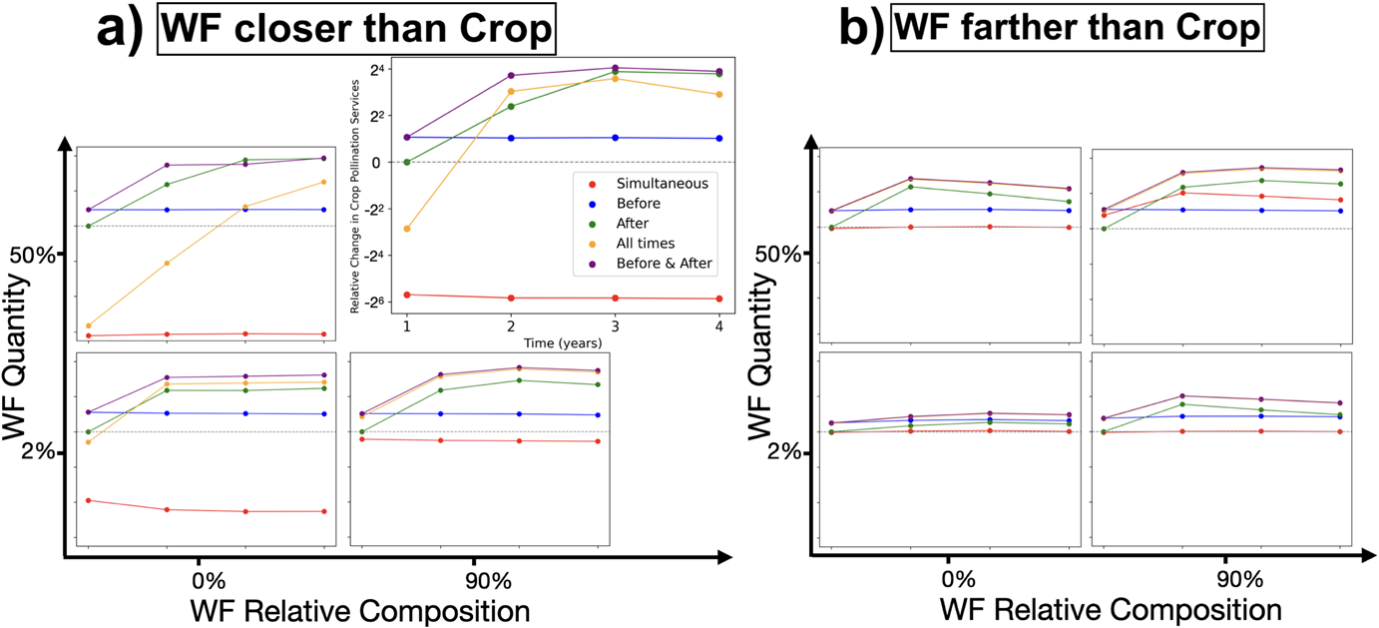
*Relative Change in Crop Pollination Services* as a function of the year since wildflower (WF) enhancements are added. Results shown for two locations, WFs closer to and farther from (a) and b) panels, respectively) the nest location than the crop; two *Relative Quantity WF* (*RWF*), 50% and 2% of the crop flower quantity (top and bottom row of each panel, respectively), and two *Relative Compositions* (*RC*, see (3.17)), 0% (same) and 90% (nearly completely different) (left and right columns of each panel, respectively). All subplots have the same axes and legend as the top right subplot (enlarged). Each color corresponds to a different blooming time for the WFs (specified in the figure legend). Other parameters: *Relative Background Flowers* is uniform and equal to  25 ml/day of nectar and 10 g/day of pollen throughout the grid. The crop is located 0.6 km from the nest in both cases, while the WF patch is positioned at the nest itself in (a) and 0.85 km away in the opposite direction of the crop in (b). The crop produces $\frac{|RC|}{100} \cdot 5000$ ml/day of nectar and $\frac{|1-RC|}{100} \cdot 1000$ g/day of pollen. The WF patch produces $RWF\frac{|1-RC|}{100} \cdot 5000$ ml/day of nectar and $RWF\frac{|RC|}{100} \cdot 1000$ g/day of pollen (see Eq. 3.17) (color figure online)

To answer questions (1-4), in Fig. 11, we show the *Relative Change in Crop Pollination Services* as a function of the year since the wildflower enhancement started, for (1) two different locations of the wildflowers, (2) two different wildflower quantities, (3) two different *Relative Composition*, and (4) five different blooming times. For the two different wildflower locations, we chose one where the wildflowers are closer to the nest than the crop, and one where the wildflowers are further away from the nest than the crop. In both cases, the crop is kept at the same distance from the nest.

This figure leads to the following findings:

- Wildflowers blooming after the crop increases pollination over a multi-year horizon due to increased number of colonies, whereas planting wildflowers that bloom before the crop increases pollination in the current year due to increased number of pollinators in time for crop bloom. Wildflowers blooming simultaneously normally causes competition for pollinators and a decrease in crop pollination services. Planting wildflowers that bloom before and after the crop is therefore a consistently beneficial practice.
- Planting farther from the nest than the crop or in smaller quantities prevents possible drawbacks, but also decreases possible benefits. The optimal quantity and location depends on the *Relative Composition* under consideration.
- Planting wildflowers with higher *Relative Composition* is best, but this is sometimes impossible in practice, since only when the crop is deficient in some way can a wildflower patch compensate for its deficits.
- The best scenario overall is when wildflowers are planted close to the nest, in large quantities, with a high *Relative Composition*, and bloom both before and after the crop blooms. The worst scenario overall is that where wildflowers are planted close to the nest, in large quantities, with low *Relative Composition*, and bloom simultaneously with the crop.

## Discussion

In this work, we present a mechanistic modelling framework to predict the population dynamics of a bumble bee colony at the nest, the spatial distribution of the foragers in a landscape, and the interaction between the two. Our novel combination of DEB for a colony and MaxEnt for spatial movement allows us to explore the interplay between colony dynamics and spatial movement from a new perspective based on entropy-energy balance.

One of the key strengths of our approach is that we are able to include a fairly high level of biological detail (movement, colony dynamics) for a relatively low computational cost. The colony-level DEB component is essentially a system of Ordinary Differential Equations (ODEs) that describes population growth based on detailed energy usage. This approach is readily accessible to many basic numerical methods and its computational cost is independent of the population size under consideration. This computational efficiency gives our model an advantage with respect to computationally-intense agent-based models such as those in Becher and Grimm (2016); Becher et al. (2018). The MaxEnt component for the spatial distribution is able to generalize the predictions of the Ideal Free Distribution to non-optimal foragers and other relevant scenarios (Capera-Aragones et al. 2023) without significantly increasing the computational cost, as is true for more classical approaches based on PDEs or agent-based modelling; the MaxEnt formulation is based on a transcendental equation, which is straightforward to solve using basic computational methods. We can therefore easily explore in detail the basic relationships between the many parameters affecting colony success and crop pollination services, such as energy flows, distance to resource, and differences between the nutritional content of crop and wildflower resources. In particular, we can vary multiple parameters simultaneously, and quickly determine how these different factors interact to affect colony success and pollination services. Furthermore, the model variables and parameters represent physically intuitive, measurable quantities, holding the potential for quantitative data validation.

In this paper we explored fairly simple landscapes, to demonstrate the effectiveness of the model and some of the interesting relationships that it reveals. Consider, for example, Fig. 10. Previous empirical work has shown that the addition of wildflower patches can be better or worse for crop pollination services (Blaauw Brett and Isaacs 2014; Buhk 2018; Delphia et al. 2022; Nicholson and Koh 2017), but has not been able to clearly identify the source of this contradiction. Modelling work in one spatial dimension (Capera-Aragones et al. 2021, 2022) or on specific agricultural landscapes (Haussler et al. 2017) has led to insights into what parameters might be responsible for the contradiction. With our model, we are able to obtain results such as those shown in Fig. 10, where we simultaneously vary previously-identified parameters as well as new ones, and do so at a fine resolution. We can thus provide a more nuanced description of the direction of the wildflower-crop pollination relationship than has been possible with previous studies.

We used the model to predict colony population dynamics under a number of landscape challenges related to the amount and density of pollen and nectar resources on the landscape, and their distance to the nest. We then used the model to predict crop pollination services over multiple years as a function of the characteristics of wildflower enhancements placed in the landscape. Our DEB/MaxEnt approach allowed us to investigate a wide variety of landscape and floral resource characteristics, within one consistent modelling framework, and over both short and long time frames. In particular, we show that pollen and nectar limitation each differently affect colony structural energy, that heating costs can lead to extinction after the early bloom of a mass-flowering crop, that growth of structural energy varies with minimum foraging distances for pollen and nectar, and that the ideal wildflower patch location and nutritional density vary in highly nontrivial ways depending on a host of parameters including, e.g., the intensity with which the landscape is farmed, the time frame considered, the relative blooming periods of the crop and wildflower resources, and the relative composition of the two floral resources.

In future work, this model could be applied to landscapes that are more complex, yet still highly flexible (Capera-Aragones et al. 2023), and could be modified to include the effects of memory on the distributions of foragers. Unlike honey bees, bumble bees are not able to communicate the precise location of specific resources, but experienced bees return repeatedly to high value sites (Dornhaus and Chittka 2004), and this behaviour certainly affects the spatial distribution of the foraging population (Capera-Aragones et al. 2021, 2022).

With future empirical work, key model parameters can be refined and improve the accuracy of the quantitative predictions of the model. In particular, measurements of the colony response (pollen, nectar, or number of foragers) to critical situations such as the ones simulated in Sect. 3.1 would be helpful for model parametrization.

One of the major simplifications of our model is the assumption that all of the nests are found in one central location. This assumption is plausible in intensively farmed landscapes, which often have few nesting sites (i.e., sites that are both suitable for nesting and that remain undisturbed by farming operations throughout the summer), and those remaining few may be localised in a relatively small area. These are precisely the landscapes where adding wildflower patches can have the greatest benefit to pollination services. The conventional standard for beauty in a flower garden mean that bumble bee gardens often provide only floral resources and not nesting sites. Our assumption, therefore, that the nesting location is separate from the wildflower patches is consistent with a worst-case scenario, where wildflower patches contribute only to floral resources. For other landscapes, the single nest location assumption is clearly a strong departure from reality, and further work is needed. With sufficient computational resources, the model we present here could be extended to include multiple nest locations.

One aspect of colony structural energy that we did not address is the wax structures built by the bees. There is currently only minimal information available regarding the amount of energy invested in wax by eusocial bumble bee colonies. Most beeswax studies focus on honey bee colonies, which are known to expend considerable colony resources into building wax honeycombs. These structures are needed for raising the brood and for storing pollen and honey. Bumble bee wax is similar to honey bee wax, but the structures built are more temporary, as they only need to last for one season, and only minimal amounts of pollen and honey are stored. We therefore assumed that we could ignore the structural energy of bumble bee colony wax for this first model, and have left its inclusion as a matter for future research.

Although here we make a particular use of the model to predict the change in pollination services of crops after wildflower enhancements, the model can be used to predict the population dynamics and spatial distribution of bumble bees in many other landscape scenarios. In addition, the model could be adapted to account for particular features of interest, such as environmental temperature dependence on colony growth and survival. Indeed, the inclusion of the DEB approach in our model means that it can be used to predict colony dynamics under future climate scenarios (Molnar et al. 2010), which is a key area of need in bumble bee and pollination modelling (Rouabah et al. 2024). The model could also be adapted to other species of pollinators other than bumble bees, or more in general, any species behaving like a super-organism.

## Data Availability

The Python code used for the simulations is available on GitHUB at https://github.com/paucapera/DEB_bees/commit/1c947424ff4e83b2aa706d4cfb5dc5959a4401e1.

## Footnotes

1 We use the term “population dynamics" in the mathematical modelling sense, i.e., to mean “changes in the number of foraging bees", regardless of whether they come from one colony or many.
